# Henipavirus zoonosis: outbreaks, animal hosts and potential new emergence

**DOI:** 10.3389/fmicb.2023.1167085

**Published:** 2023-07-17

**Authors:** Hongzhao Li, Ji-Young V. Kim, Bradley S. Pickering

**Affiliations:** ^1^National Centre for Foreign Animal Disease, Canadian Food Inspection Agency, Winnipeg, MB, Canada; ^2^Department of Medical Microbiology and Infectious Diseases, College of Medicine, Faculty of Health Sciences, University of Manitoba, Winnipeg, MB, Canada; ^3^Department of Veterinary Microbiology and Preventive Medicine, College of Veterinary Medicine, Iowa State University, Ames, IA, United States

**Keywords:** henipavirus, Hendra virus, Nipah virus, epidemiology, transmission, animal host, livestock, henipa-like virus

## Abstract

Hendra virus (HeV) and Nipah virus (NiV) are biosafety level 4 zoonotic pathogens causing severe and often fatal neurological and respiratory disease. These agents have been recognized by the World Health Organization as top priority pathogens expected to result in severe future outbreaks. HeV has caused sporadic infections in horses and a small number of human cases in Australia since 1994. The NiV Malaysia genotype (NiV-M) was responsible for the 1998–1999 epizootic outbreak in pigs with spillover to humans in Malaysia and Singapore. Since 2001, the NiV Bangladesh genotype (NiV-B) has been the predominant strain leading to outbreaks almost every year in Bangladesh and India, with hundreds of infections in humans. The natural reservoir hosts of HeV and NiV are fruit bats, which carry the viruses without clinical manifestation. The transmission pathways of henipaviruses from bats to humans remain poorly understood. Transmissions are often bridged by an intermediate animal host, which amplifies and spreads the viruses to humans. Horses and pigs are known intermediate hosts for the HeV outbreaks in Australia and NiV-M epidemic in Malaysia and Singapore, respectively. During the NiV-B outbreaks in Bangladesh, following initial spillover thought to be through the consumption of date palm sap, the spread of infection was largely human-to-human transmission. Spillover of NiV-B in recent outbreaks in India is less understood, with the primary route of transmission from bat reservoir to the initial human infection case(s) unknown and no intermediate host established. This review aims to provide a concise update on the epidemiology of henipaviruses covering their previous and current outbreaks with emphasis on the known and potential role of livestock as intermediate hosts in disease transmission. Also included is an up-to-date summary of newly emerging henipa-like viruses and animal hosts. In these contexts we discuss knowledge gaps and new challenges in the field and propose potential future directions.

## Introduction

Hendra virus (HeV) and Nipah virus (NiV) are highly virulent, prototypic members of the *henipavirus* genus in the *Paramyxoviridae* family (Chua et al., 1999; Nordin, 1999; Chua et al., 2000; ICTV, 2023). The viruses were named after outbreak locations where they were first isolated, the suburban town of Hendra in Australia (Murray et al., 1995; nsw.gov.au, 2023) and the village of Kampung Sungai Nipah in Malaysia (Mohd Nor et al., 2000), respectively. For the purpose of this review, the term “henipaviruses” is used as traditionally represented for both HeV and NiV. A number of newly emerging viruses closely related to HeV and NiV are collectively referred to as “henipa-like viruses” here, including those that have been officially classified into the *henipavirus* genus (ICTV, 2023) and those whose potential classification into the genus remains to be confirmed. For the time being, these henipa-like viruses have largely uncertain zoonotic and pathogenic potential and are not (yet) established as major public health threats. However, despite the infancy of their research, signs of potentially far-reaching impact are emerging. Therefore, while the prototypic henipaviruses constitute the core of this review, we also summarize and provide perspectives on the new henipa-like viruses in terms of both challenges and opportunities.

The virions of henipaviruses are enveloped and pleomorphic with variable sizes from 120 to 500 nm and have an unsegmented single-stranded RNA genome of negative polarity (Chua et al., 2000). The henipaviral genome contains six protein-coding genes, nucleoprotein (N), phosphoprotein (P), matrix protein (M), fusion protein (F), glycoprotein (G) and large protein/polymerase (L), organized in the order of 3’-N-P-M-F-G-L-5′. The G protein was recently renamed as the receptor binding protein (Rima et al., 2019). However, we keep the traditional name for simplicity in the context of this review. The P gene also allows for the expression of three accessory proteins, V, W and C, through mRNA editing or alternative open reading frames. The genome lengths of henipaviruses (~ 18.2 kilobases) are longer (by ~15%) than those of other paramyxoviruses (~ 15.5 kilobases), largely due to the long 3′ untranslated regions of the N, P, F and G mRNAs (Eaton et al., 2006; Madera et al., 2022; Bruno et al., 2023).

In humans and several animal species, the major pathological consequence of henipaviral infection is a severe acute systemic vasculitis in many major organs, prominently in the brain and lung. This often leads to fatal neurological or/and respiratory disease, which also includes long-term relapsing encephalitis (from several months to over a decade following infection) (Playford et al., 2010; Wong and Ong, 2011; Wong and Tan, 2012; Broder et al., 2013; Abdullah and Tan, 2014; nsw.gov.au, 2023). Henipaviruses are classified as biosafety level 4 pathogens due to extremely high case fatality rates (CFR), up to 70–100% in some of the recent outbreaks, and the absence of licensed vaccines or therapeutics for human use (Skowron et al., 2021; Quarleri et al., 2022; Bruno et al., 2023). It should be noted that Equivac® HeV, a subunit vaccine based on a recombinant soluble and oligomeric form of the HeV G glycoprotein, was released in 2012 for immunization of horses in Australia. It is the first licensed (veterinary) vaccine against a biosafety level 4 agent (Broder et al., 2013). However, a human vaccine currently remains unavailable. The World Health Organization has listed henipaviruses as priority pathogens of epidemic and pandemic potential in urgent need for research and development (Sweileh, 2017; Mehand et al., 2018; WHO, 2023a,b,c).

## HeV outbreaks: Australia

Since its emergence in 1994 in Australia, HeV has been causing sporadic infections in horses in most of the subsequent years, and on an annual basis since 2006, with the most recent case reported in July 2022 (Murray et al., 1995; Halpin and Rota, 2014; Wang et al., 2021; nsw.gov.au, 2023; qld.gov.au, 2023). Human infections have been linked to close contact with sick horses. To date, there have been 88 confirmed cases and 20 suspected cases of HeV infection in horses and seven confirmed cases in humans (nsw.gov.au, 2023; qld.gov.au, 2023). Although the case numbers have so far been small, HeV infection shows a concerning pattern of continued and frequent occurrences as mentioned above and a strikingly high pathogenesis. The disease in horses is typically characterized by acute central neurological symptoms and sudden death. Elevated heart rate and respiratory rate have been frequently observed (qld.gov.au, 2023). Based on available data the CFR in horses was estimated to be 80% (Yuen et al., 2021). The precise CFR was not possible to determine as horses with a positive diagnosis of HeV while remaining alive had to be euthanized according to the regulatory policy (Yuen et al., 2021). The human cases demonstrated a CFR of 57% (4/7), mostly underlain by fatal encephalitis (nsw.gov.au, 2023).

Apart from the established HeV prototype, or genotype 1 (HeV-g1), a novel variant, or genotype 2 (HeV-g2), was detected in Australia in horses that suffered acute illness with signs of HeV infection as well as in fruit bats (Wang et al., 2021; Annand et al., 2022; Peel et al., 2022; Taylor et al., 2022). The two genotypes share a 84% or 83.5% nucleotide identity in genomic sequences (Wang et al., 2021; Annand et al., 2022). The sequence divergence accounted for the previous failure to detect HeV-g2 by traditional PCR targeting HeV-g1 (Wang et al., 2021; Annand et al., 2022). At the protein level, HeV-g2 exhibits an 82.3–95.7% (mean 92.5%) amino acid identity to HeV-g1 (Annand et al., 2022). Consistent with the higher degree of conservation in protein sequences, the G proteins of the two genotypes share a conserved receptor tropism, and broadly neutralizing monoclonal antibodies to the F and G proteins were found to potently neutralize both HeV-g1 and HeV-g2 (Wang et al., 2022). These data suggest that the antibody post-exposure prophylaxis and equine vaccine against HeV-g1 should be effective against HeV-g2 (Annand et al., 2022; Wang et al., 2022).

## NiV outbreaks: Malaysia-Singapore (NiV-M) and Bangladesh-India (NiV-B)

NiV, initially known as Hendra-like virus, was discovered during the 1998–1999 outbreak in farmed pigs and humans in Malaysia (Chua et al., 1999; Paton et al., 1999; CDC, 1999a,b; Chua et al., 2000; Mohd Nor et al., 2000; Lam and Chua, 2002; Chua, 2003; Kummer and Kranz, 2022). The spread via transport of infected pigs also led to a small number of human cases in Singapore More than a million Malaysian pigs were culled to control the epidemic, a huge loss to the agriculture industry. Infected pigs largely lacked clinical disease while some showed neurological and respiratory symptoms (Mohd Nor et al., 2000). The mortality was low (less than 1–5%) except in piglets (approximately 40%) (Mohd Nor et al., 2000). Human infections, apparently through direct contact with contaminated tissues/body fluids of infected pigs, were characterized by severe febrile encephalitis with a CFR of 38% (106/276) (Chua et al., 2000; Goh et al., 2000; Chong et al., 2002; Lewis and Pickering, 2022).

Since 2001, in Bangladesh and its neighboring regions of India, NiV outbreaks or isolated transmission events have been reported almost every year, mainly during the winter months, and have resulted in hundreds of infections in humans (Hsu et al., 2004; Chadha et al., 2006; Halpin and Rota, 2014). A current NiV outbreak in Bangladesh, according to media reports, has been announced by health authorities on January 29, 2023 (risingbd.com, 2023), and eight people have died out of 11 identified cases by February 13, 2023 (bdnews24.com, 2023; outbreaknewstoday.com, 2023; thedailystar.net, 2023). With the winter season ongoing at this moment, more cases may occur from this outbreak. The winter seasonality of NiV (and HeV) cases remains to be understood, which might be multi-factorial (Martin et al., 2018; McKee et al., 2021). Variations in NiV spillover were shown to correlate with winter temperatures, which may affect the physiology and viral dynamics in bats and the behaviors of bats and humans (Cortes et al., 2018; McKee et al., 2021). The secretion pulses of HeV were found to be higher in winter (Field et al., 2015). We speculate that this may also be the case for NiV, and in addition NiV shed into the environment may be more stable with infectious activity lasting longer under the lower temperatures in winter. These would increase the chance of infection in exposed hosts.

The latest NiV case in India to our knowledge was documented in a report by the World Health Organization (WHO, 2021) and in a research publication (Yadav et al., 2022). The infections in Bangladesh and India differed in several aspects from the original 1998–1999 Malaysia outbreak: No animal host susceptible to clinical disease was identified with role of viral transmission to humans; The CFRs were significantly higher, ranging from approximately 70% to above 90%, up to 100% (Skowron et al., 2021); A majority of cases resulted from human-to-human transmissions (absent in the Malaysia outbreak), which raises serious concern of potential larger-scale outbreaks or even pandemic in humans; And severe respiratory disease, infrequently seen in the Malaysia outbreak, was found in over 60% of these infections, and might be a contributing factor to the transmission and mortality patterns (Luby et al., 2009b; Halpin and Rota, 2014; Kasloff et al., 2019). Consistent with their distinct epidemiological and clinical features, phylogenetic analysis showed that NiV strains from Malaysia and those from Bangladesh and India represent two separate genetic lineages, defined as NiV-M and NiV-B, respectively (Lo et al., 2012). The NiV-M genotype also includes a Cambodia isolate from bat urine (Reynes et al., 2005; Lo et al., 2012).

## NiV-like virus outbreak: Philippines

A possibly new type of NiV or NiV-like virus was reported from a 2014 outbreak in the Philippines (Ching et al., 2015). The outbreak involved 17 human cases, with neurological and influenza-like symptoms and a CFR of 53% (9/17), and 10 fatal cases of horses, characterized by sudden death and neurological symptoms (CFR in horses unavailable with total infection number unknown). It should be noted that there was no clear diagnosis made regarding the cause of death in horses, presumably it was NiV but this was not firmly established. Epidemiological evidence suggested possible horse-to-human and human-to-human transmissions. Serological and molecular data supported that the virus causing this outbreak was a henipa-like virus antigenically and genetically. A sequence read (71 bp) of the viral genome corresponding to the P gene of NiV had 99%, 94–96 and 80% identity with NiV-M, NiV-B and HeV strains, respectively. Further attempts to amplify/sequence additional genomic sequences and isolate the virus were unfortunately unsuccessful (Ching et al., 2015). It is unknown if the same or similar NiV-like viruses are still circulating in their natural reservoir host(s) in the Philippines (or elsewhere). To date there has been no new report of subsequent detection of such viruses or any follow-up investigation into their presence. Molecular and serological surveillance studies of henipaviral infection in wildlife and livestock are encouraged to be carried out in endemic regions, such as in bats and horses in the area of the Philippine outbreak.

## Natural hosts for the transmission of henipaviruses

HeV and NiV, like several other major pathogens threatening global public health, are emerging RNA viruses from bats (Tian et al., 2022). Several species of Old World fruit bats (also known as flying foxes) from the genus *Pteropus*, family *Pteropodidae* are the major known reservoir hosts of henipaviruses (Drexler et al., 2009; Halpin et al., 2011; Geisbert et al., 2012; Middleton and Weingartl, 2012; Kessler et al., 2018; Tian et al., 2022). Of interest, most filoviruses, including Ebola and Marburg-related viruses, were identified in other genera of the same bat family, *Pteropodidae* (Tian et al., 2022), while SARS-CoV-2-like coronaviruses were recently found in bats of another family, *Rhinolophidae* (Temmam et al., 2022; Tian et al., 2022). The Pteropus bats have a geographic distribution involving eastern Africa (Madagascar island), Asia, Australia and the Pacific islands (Iehlé et al., 2007; Gurley et al., 2017; Kessler et al., 2018), illustrating a broad area at potential risk for henipavirus disease.

Henipaviruses can be transmitted both among bats and in spillover events to other animals and humans (Quarleri et al., 2022).The Pteropus hosts do not exhibit any evident disease with natural or experimental infection by henipaviruses (Middleton and Weingartl, 2012; Halpin and Rota, 2014; Quarleri et al., 2022). The long-term coexistence in harmony between the viruses and their reservoir hosts may represent a relative equilibrium reached during co-evolution (Halpin and Rota, 2014). Transmission of henipaviruses to humans is often bridged by an intermediate animal host, which amplifies and spreads the viruses to humans. Candidate intermediate hosts can be livestock that interact with both the reservoir hosts and humans (Kummer and Kranz, 2022). Horses and pigs are clearly established intermediate hosts for the HeV outbreaks in Australia and NiV-M epidemic in Malaysia and Singapore, respectively (Halpin and Rota, 2014; Kummer and Kranz, 2022). It is unknown whether other livestock, such as domestic ruminants (goats, sheep and cattle), can serve as intermediate hosts (Skowron et al., 2021; Kummer and Kranz, 2022).

During the NiV-B outbreaks in Bangladesh and India, although the spread of infection was largely through human-to-human transmission, the primary transmission route linking the bat reservoir to the initial human infection case(s) was not known, and no intermediate host was established. Outbreaks in pigs, which had previously contributed to human infections in Malaysia and Singapore, were not seen here (Luby et al., 2009a). In a recent experimental infection study conducted in our lab, NiV-B did not lead to any clinical signs in infected pigs and viremia was not detectable at any sampling time point from short-term and long-term time series. However, infectious viruses were isolated from several pig tissues and nasal washes (Kasloff et al., 2019). A “silent” infection without clinical signs could pose significant risk of viral transmission. In addition, comparing across the past henipavirus outbreaks, the data together suggest that genetic divergence in henipaviruses can shift their biological behaviors concerning host tropism, pathogenicity, amplification and transmission with epidemiological impact.

Human consumption of date palm sap suspected of henipaviral contamination from bats was found to correlate with risk of NiV-B infection and has been proposed as the most common pathway of NiV-B transmission from bats to humans (Luby et al., 2009a; Luby and Gurley, 2012; Rahman et al., 2012). Infrared wildlife photography showed that Pteropus bats frequently visit date palm trees, lick the sap stream and urinate near the sap collection pot (Luby and Gurley, 2012; Rahman et al., 2012). Skirt barriers of bamboo (or other materials) were tested and found effective to impede bat access to date palm sap (Khan et al., 2012; Nahar et al., 2014). The hypothesis was further supported in a laboratory setting where NiV was transmitted from artificial palm sap to Syrian hamsters (de Wit et al., 2014). To date no NiV has been isolated directly from date palm sap (Rahman et al., 2012). It should be noted that viral isolation attempts have only been made on sap samples collected weeks after an outbreak and the timing may not be suitable for the purpose since Pteropus shedding of NiV is intermittent (Luby and Gurley, 2012). In addition, so far there has been no report whether the prevention of sap contamination by bats using skirt barriers leads to reduced outbreak cases. Additional studies are needed to confirm the role of sap consumption in viral transmission. While no direct evidence has proved the correlation between sap consumption and NiV-B outbreak to be causal in the natural transmission setting, it is an open possibility that alternative mode(s) of transmission may exist and unidentified intermediate host(s) could serve as the transmission route or an additional transmission route between bats and humans (Skowron et al., 2021).

## A diversity of undetermined transmission mechanisms

Henipaviral transmissions concerning human infections can occur from bats to intermediate hosts, from intermediate hosts to humans, potentially from bats to humans without intermediate hosts or from humans to humans. These events are commonly featured by close/direct contact with infected hosts or the proximity to bat presence, which has been well supported by scientific observations. However, specific details of the transmission mechanisms remain to be determined while a number of potential modes and vehicles of transmission have been proposed (Luby et al., 2009a; Gazal et al., 2022; Bruno et al., 2023).

Body fluids and excretions from henipavirus-infected bat, horse, pig or human hosts, such as saliva, urine and feces, or materials with these contaminants such as fruits partially eaten by bats and date palm saps in contact with bats have been believed to be vehicles of viral transmission. This applies to the handling or consumption of meat from infected pigs or horses as well (Luby et al., 2009a; Gazal et al., 2022; Bruno et al., 2023). It was also mentioned that direct shedding to receptive animals or inhalation of aerosol NiV virions could be possible modes of transmission from bats to animals (Bruno et al., 2023), which however have not yet been scientifically tested. As respiratory symptoms were indicators of NiV-B infectivity (Nikolay et al., 2019), droplets from coughing and sneezing could be a vehicle of transmission (Hsu et al., 2004; Gurley et al., 2007; Bruno et al., 2022). Finally, semen samples from a survivor of NiV infection in India were tested positive for NiV RNA on days 16 and 26 post onset of illness (Arunkumar et al., 2019). It will be interesting to test the possibility of NiV persistence in semen and transmission through the sexual route, which is true for Ebola and Zika viruses (Arunkumar et al., 2019). Part of the test should be the isolation of infectious NiV virus from semen.

These hypotheses warrant further research as they are both theoretically feasible and have been suggested or supported to some extent by outbreak observations. Their determination will require in-depth epidemiological investigations and controlled laboratory studies.

## A wider range of potential hosts for henipaviruses than the known

The host receptors for henipaviruses, ephrin-B2 and ephrin-B3, are highly conserved among mammalian species, which in theory allow the viruses to infect a broad range of hosts. Consistent with this, apart from the known natural hosts bats, horses and pigs, many small animals were able to be experimentally infected by henipaviruses supporting viral replication (Weingartl et al., 2009; Geisbert et al., 2012; Tian et al., 2022). These include guinea pigs, golden hamsters, cats, ferrets and African green monkeys. However, the levels of viral amplification and clinical features vary greatly among these animals, although their viral receptors share similar efficiency in mediating viral entry (Tian et al., 2022). In addition, mice were resistant to henipaviral infection despite having similar functional viral receptors. These suggest that host factors other than the receptors also contribute to the outcomes of henipavirus-host interactions (Tian et al., 2022). Nevertheless, the current data point to the possibility that henipaviruses have the capacity to infect a diverse range of animal hosts. Therefore, it is an open possibility that additional, as-yet-unidentified animal hosts other than the known natural hosts could transmit henipaviruses.

## Domestic ruminants as potential intermediate hosts

Domestic ruminants including goats, sheep and cattle constitute significant part of agriculture. They have worldwide distributions in large population numbers, which also involve areas overlapping with habitats of henipaviral reservoir hosts. Particularly, for example, the estimated numbers of goats, sheep and cattle are 14.8 million, 1.9 million and 25.7 million, respectively, in Bangladesh (Banglapedia, 2023), and 148.89 million, 74.26 million and 193.46 million, respectively, in India (pib.gov.in, 2023). Many of them are constantly exposed at the interface between a farming area and bat territory and may well be in a position to bridge henipaviral transmission to humans. Little has so far been studied, however, concerning the potential role of these animals in zoonotic spillover of henipaviruses, especially NiV-B, for which no intermediate hosts have been identified yet. The lack of attention is likely due to the absence of disease outbreaks in ruminants at large scales as seen in pigs during the Malaysia and Singapore NiV-M epidemic. This may possibly reflect difference between NiV-B and NiV-M in host tropism, pathogenicity or/and transmissibility. Another conceivable hypothesis is based on the difference between the ways of raising livestock in the Malaysian and Bangladesh outbreak areas (Bruno et al., 2023). In the Malaysian case, the rapid and widespread NiV transmission was underlain by dense pig farming. In contrast, in Bangladesh livestock are managed in small groups sparsely populated, which could have limited the scale of possible transmissions.

Several signs suggest potential exposure of ruminants to henipaviruses and their possible involvement in viral transmission to humans. It was reported that in 2004 two goats owned by a Bangladesh family became ill with symptoms including “fever, difficulty walking, walking in circles, and frothing at the mouth” and both died. Within 2 weeks following the goats’ death, a boy from the family, who had played with the goats developed encephalitis and tested positive for NiV antibodies (Luby et al., 2009a). This raises the possibility that goats may play a role in transmitting NiV-B, and disease can develop in infected goats. The goat disease is probably a small-scale, low-frequency event, which may often be overlooked or neglected. However, due to human-to-human transmission of NiV-B, a single spillover case from goats could kindle an outbreak in humans. Whether infection events with similar characteristics could also occur in other animals such as cattle and sheep is an open question. In addition, antibodies to NiV glycoprotein were detected in both goats and cattle in a Luminex-based serological assay where sheep were not tested (Chowdhury et al., 2014). Finally, outbreak surveys identified a correlation between NiV-B cases and contact with sick cows (Hsu et al., 2004; Luby et al., 2009a).

These possibilities need to be observed further in epidemiological investigations and tested in controlled experiments. Knowledge on the potential role of ruminants as amplifying hosts for henipavirus transmission, which is currently lacking, is of great veterinary and public health importance, considering the vast quantity of potential interactions between humans and henipavirus reservoir hosts connected through ruminants at the ecological interface. New research findings on whether or not ruminants do play such a role will clearly provide guidance for governments to prioritize targets for prevention, surveillance, diagnosis and further research.

## Newly emerging henipa-like viruses and animal hosts

The genus *Henipavirus* was initially created in the family *Paramyxoviridae* following the identification of the two prototype viruses, HeV and NiV (Chua et al., 2000). With the continued discovery of new henipa-like viruses, the genus has currently included three more members, Cedar virus (CedV), Ghana virus (also called Kumasi virus or Ghanaian bat virus) and Mòjiāng virus (MojV) (Amarasinghe et al., 2017), and further expansion is expected to include additional henipa-like viruses that have recently been reported.

CedV was isolated from urine samples collected in a Pteropus bat colony and named after the sampling location, Cedar Grove in Australia (Marsh et al., 2012). The virus shares key features with prototypic henipaviruses including nearly identical genomic size, at 18,162 nucleotides (nt), and coding structure, in the order of 3’-N-P-M-F-G-L-5′, as well as antigenic cross-reactivity, and was thus classified into the *henipavirus* genus (Marsh et al., 2012). However, it has several distinct characteristics. CedV is currently considered non-pathogenic (Marsh et al., 2012; Lieu et al., 2015; Laing et al., 2018; Schountz et al., 2019). No outbreaks in humans or livestock have been reported. Experimental CedV infection in ferrets, guinea pigs or hamsters, small animal models known to be susceptible to henipavirus disease, does not cause clinical disease despite viral replication and production of neutralizing antibodies (Marsh et al., 2012; Schountz et al., 2019). The isolation of a non-pathogenic (or less-pathogenic) virus closely related to the highly pathogenic henipaviruses offers a powerful tool for targeted comparative studies into the determinants of differences among these viruses. Such studies will bring valuable insights into the biology of henipaviruses and identify novel targets for the development of vaccines and antivirals. CedV was originally isolated in a containment level 4 laboratory and the live infectious virus is unavailable for use in a lower containment setting since any material to be removed from containment level 4 has to be fully inactivated. For that reason, recombinant CedVs were generated outside containment level 4 using reverse genetics approach, including those expressing a green fluorescent protein or luciferase reporter or G and F proteins of HeV or NiV. These are contributing to basic research and the development of compound and monoclonal antibody antivirals (Laing et al., 2018; Amaya et al., 2021; Doyle et al., 2021). On the genetic level, a major difference between CedV and prototypic henipaviruses lies in the coding strategy of the P gene. In CedV, the P gene does not encode V or W proteins, which are both antagonists used by henipaviruses against host innate antiviral responses. Additionally, for host cell entry CedV uses ephrin-B2, ephrin-B1, ephrin-A2 and ephrin-A5 but not ephrin-B3, while both ephrin-B2 and ephrin-B3 are used by the pathogenic henipaviruses. The failures of CedV to produce V and W proteins and to use the ephrin-B3 receptor have been suggested to be contributing factors to its nonpathogenic phenotype in the animal infection models (Marsh et al., 2012; Lieu et al., 2015; Laing et al., 2018; Schountz et al., 2019). Whether these proposed mechanisms are essential or sufficient for the determination of pathogenicity phenotypes will need to be further addressed by extended revere genetics studies (with mutagenesis or recombination between CedV and prototypic henipaviruses that may cause changes in pathogenicity). Experiments with potential to generate infectious viral mutants of undetermined pathogenicity such as viral rescue or propagation in cell culture would need to be performed in containment level 4 and gain duel use institutional approval.

Ghana virus has been named after the location (Kumasi city, Ghana) where the sequence of its RNA genome was first identified (Drexler et al., 2009; Drexler et al., 2012). Although henipavirus disease outbreaks have so far only been attributed to reservoir bats from the *Pteropus* genus and reported from endemic areas within Australia and Southeast/South Asia, serological and molecular evidences suggest the existence of henipa-like viruses beyond these boundaries. African fruit bats of the *Eidolon* genus (also in the *Pteropodidae* family) may be an additional host type. These bats are found in continental Africa, which has no Pteropus bats, as well as Madagascar. Antibodies to henipaviruses were detected in both Pteropus and Eidolon bats in Madagascar (Iehlé et al., 2007), Eidolon bats in Ghana (Hayman et al., 2008) and Eidolon bats and humans in Cameroon (Pernet et al., 2014b). In all these serological studies, the antibodies displayed neutralization activity against henipaviruses. Genetic sequences corresponding to potential new virus species in close phylogenetic relationship to henipaviruses were identified in African bats (Drexler et al., 2009, 2012). These are currently known to involve five bat genera from the *Pteropodidae* family (*Eidolon, Epomophorus, Hypsignathus, Myonycteris and Rousettus*) and five central/west African countries (Democratic Republic of the Congo, Republic of the Congo, Gabon, Central African Republic and Ghana) (Drexler et al., 2009, 2012). The full genome sequence of a representative virus (the prototype Ghana virus: originally called GH-M74a, 18,530 nt, GenBank accession number HQ660129) of these confirmed formal classification into the *henipavirus* genus (Drexler et al., 2012). Furthermore, henipa-like sequences were also found in New World bats in central and south America including Costa Rica. These bats belong to genera in other families than *Pteropodidae*, which are genus *Carollia* in family *Phyllostomidae* and genus *Pteronotus* in family *Mormoopidae* (Drexler et al., 2012). These findings were further supported by the detection of antibodies to henipaviruses in Brazilian bats (de Araujo et al., 2017).

MojV, a rodent-borne virus, is the first established member of the *henipavirus* genus that has been reported in a non-bat reservoir host, shifting the paradigm of henipavirus reservoir host range. The virus was named after the Mòjiāng County, Yunnan Province, China, where an outbreak of pneumonia disease with unknown etiology in 2012 resulted in the death of three miners who had been working in a mine cave (CFR 100%). Following the incidence, a virome survey was conducted on anal swab samples of bats, rats and musk shrews collected from the cave, leading to the identification of MojV sequence in rats (*Rattus flavipectus*) (Wu et al., 2014). MojV (GenBank accession no. KF278639) demonstrates typical genomic features of henipaviruses in size (18,404 nt) and organization (3’-N-P-M-F-G-L-5′) and in phylogenetic analysis clusters with the other four members of the *henipavirus* genus, confirming its classification into the genus (Wu et al., 2014). However, the glycoprotein of MojV lacks the ephrin binding motif, which mediates receptor recognition in prototypic henipaviruses, and does not bind ephrin-B2 or ephrin-B3 *in vitro*, suggesting a distinct, ephrin-independent host cell entry mechanism (Rissanen et al., 2017). In addition, antibodies to well-established henipaviruses were not found to cross-react with the glycoprotein of MojV. This implies that serological screening studies based on reagents derived from typical henipaviures may have missed the detection of MojV, leading to a potential underestimation of their prevalence (Rissanen et al., 2017).

Apart from the five members that have so far been formally classified into the *henipavirus* genus (ICTV, 2023), new henipa-like viruses continue to be discovered in the recent years. One of these, the Angavokely virus (AngV), was identified from a urine sample of Eidolon bats in Madagascar (Madera et al., 2022). This is consistent with the previous detection of antibodies to henipaviruses in those bats (Iehlé et al., 2007). The sequence of a nearly complete 16,740-nt genome of this virus was recovered. It displays a structural organization characteristic of the *henipavirus* genus. Protein structure modeling predicted that the glycoprotein of AngV lacks ephrin binding residues, similarly to MojV.

Following MojV, more henipa-like species have been identified in potential reservoir hosts other than bats. A molecular screening study in African rodents and shrews in Zambia identified henipa-like RNA sequences using RT-PCR primers targeting the L gene. In phylogenetic analysis one rodent sequence and seven shrew sequences clustered in close relation to the *henipavirus* genus, pointing to the potential existence of henipa-like viruses not only in rodents but also in shrews (Sasaki et al., 2014). Indeed, shrew-borne henipa-like viruses were later identified in Asia (Korea and China), Africa (Guinea) and Europe (Belgium). Among these are the Gamak virus (GAKV) and Daeryong virus (DARV), named after the locations of their origin in the Republic of Korea (Gamak and Daeryong Mountains, respectively) (Lee et al., 2021). The viruses were detected from kidney tissues of shrews belonging to the *Crocidura* genus in the *Soricidae* family and virus isolation was successful for GAKV (Lee et al., 2021). The nearly complete genomes of GAKV and DARV are approximately 18,460 nt and 19,471 nt in size, respectively. Both present a coding structure typical of henipaviruses, 3’-N-P-M-F-G-L-5′. The P gene sequence appears to encode the P, C,V and W proteins with a putative RNA editing site. Phylogenetic analysis demonstrated that GAKV and DARV are closely related to the *henipavirus* genus (the most closely related to MojV among the genus members) and share a common ancestor with MojV (Lee et al., 2021). In a more recent study, two henipa-like viruses, the Melian virus (MeliV) and Denwin virus (DewV), were detected in Crocidura shrews from Guinea and Belgium, respectively (Vanmechelen et al., 2022). Both viruses have a genomic organization characteristic of henipaviruses in the order of 3’-N-P-M-F-G-L-5′. It should be noted, however, that the sizes of both genomes are noticeably larger than the average size of prototypic henipaviruses, which are 19,944 nt in MeliV and 19,746 nt in DewV (Vanmechelen et al., 2022). This is also observed at least in DARV, while the sequencing of the GAKV and DARV genomes was described as nearly complete (Lee et al., 2021). Phylogenetic analysis indicated that MeliV and DewV are closely related to the *henipavirus* genus and cluster together with MojV, GAKV and DARV (Vanmechelen et al., 2022).

Langya virus (LayV) is the most recently reported shrew-borne henipa-like virus (Mallapaty, 2022; Zhang et al., 2022). A hospital surveillance of febrile patients with a recent history of animal exposure was conducted in the eastern Chinese provinces of Shandong and Henan. As a result, LayV was isolated from a throat swab sample of a patient from the town of Langya in Shandong. The LayV genome exhibits features typical of henipaviruses: 18,402 nt in length and organized as 3’-N-P(also encoding V/W/C)-M-F-G-L-5′. Phylogenetically, the virus clusters with *henipavirus* genus and related henipa-like viruses, with the closest relation to MojV (Zhang et al., 2022). A subsequent investigation identified 35 patients with acute infections involving LayV, of which 26 patients were infected with LayV only. The 26 patients presented a variety of symptoms with variable severities including respiratory symptoms, but there were no fatalities (Mallapaty, 2022; Zhang et al., 2022). To identify potential animal hosts, molecular survey in 25 wild small animals detected LayV RNA predominantly in shrews, supporting their potential role as a reservoir host for LayV. Serological survey in domestic animals detected antibodies to LayV in goats and dogs, suggesting that goats and dogs may be susceptible to LayV infection and could serve as potential intermediate hosts (Zhang et al., 2022).

The rodent-borne and shrew-borne henipa-like viruses appear to cluster together with one another, more closely than they do with the bat-borne viruses, and vice versa (Sasaki et al., 2014; Lee et al., 2021; Hernández et al., 2022; Vanmechelen et al., 2022). Thus, two closely related but distinct viral clades, a rodent/shrew-borne clade and a bat-borne clade, should be considered to constitute the *henipavirus* genus. The rodent/shrew-borne clade seems to be characterized by a conserved putative protein coding region (for a small transmembrane protein) between the M and F genes, which is not found in other paramyxoviruses (Vanmechelen et al., 2022).

Beyond bats, rodents and shrews, a new study pointed to the possibility that henipa-like viruses could be carried by other natural reservoir hosts. In Brazilian opossums (*Marmosa demerarae*) a henipa-like virus was detected, named Peixe-Boi virus (PBV) based on the location of sampling, the municipality of Peixe-Boi, Pará State, Brazil (Hernández et al., 2022). A partial genomic sequence of PBV (2,377 nt), corresponding to part of NiV L gene, was available and deposited into GenBank with accession number MZ615319. In phylogenetic analysis, the PBV sequence shared a common ancestry with the *henipavirus* genus and related henipa-like viruses and clustered with these viruses as a close sister branch but further away from other genera of *Paramyxoviridae* (Hernández et al., 2022).

Finally, it should be noted that among the henipa-like viruses discussed above, CedV, GAKV, LayV are virus isolates whereas the others are viral sequences and remain to be isolated as whole viruses.

**Figure 1:**
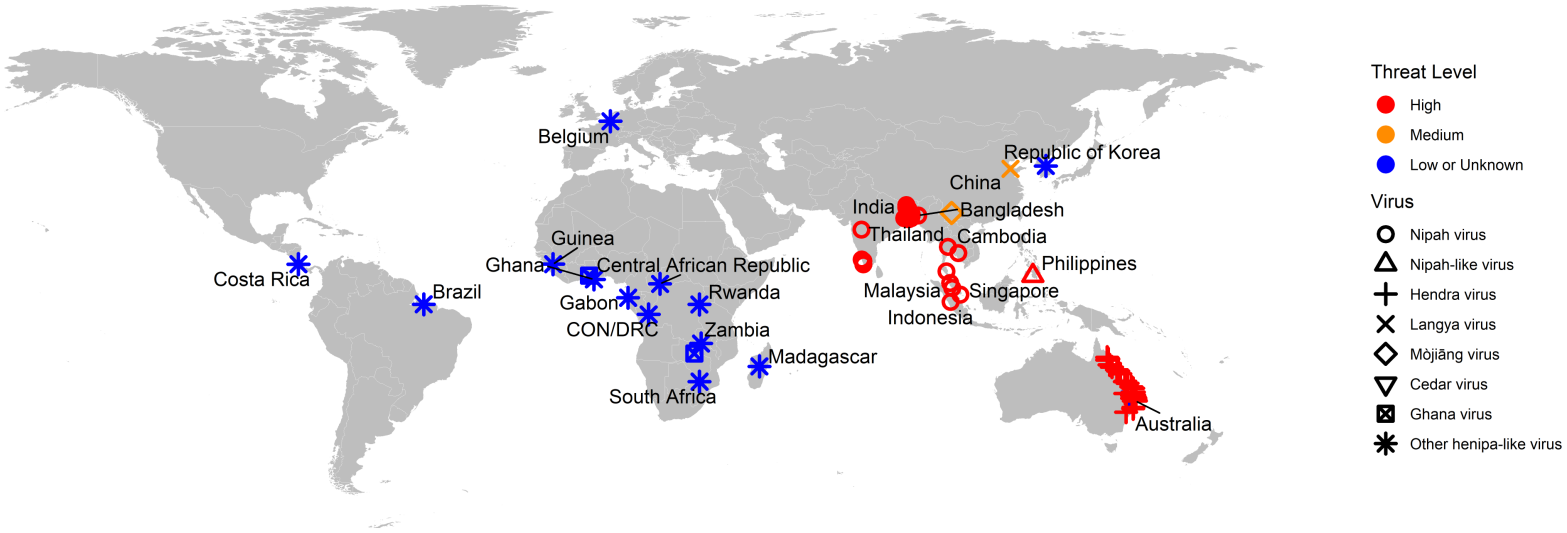
Global locations of henipaviruses and henipa-like viruses. The virus type is indicated by shape. The threat level is based on the degree of fatality in humans. CON and DRC = Republic of the Congo and Democratic Republic of the Congo, respectively.

**Figure 2:**
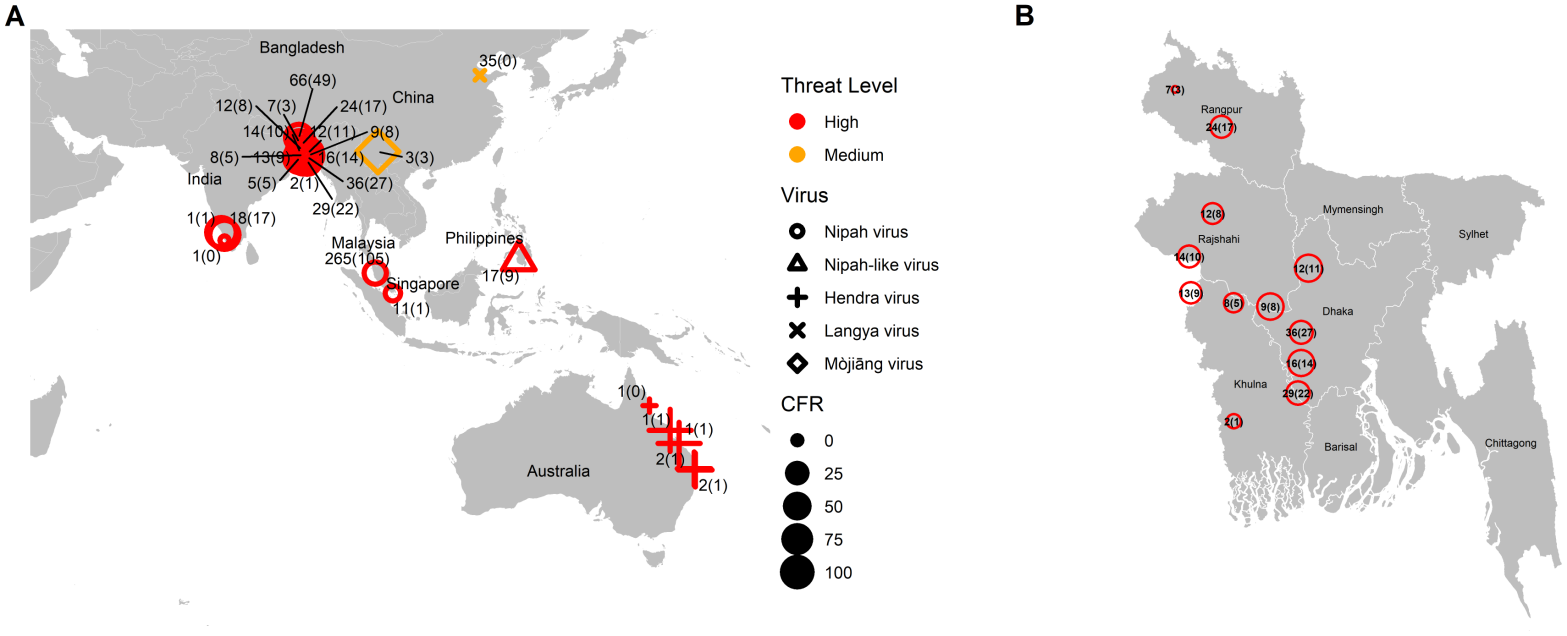
Global locations of henipavirus and henipa-like virus outbreaks in humans causing diseases/fatalities. **(A)** Global map. The shape of the symbol represents the virus type, while the size represents the case fatality rate (CFR). The number pair indicates the total number of infected with fatal cases in parentheses. The threat level is determined based on the degree of fatality in humans. **(B)** Enlarged illustration of outbreak cases in Bangladesh.

**Figure 3:**
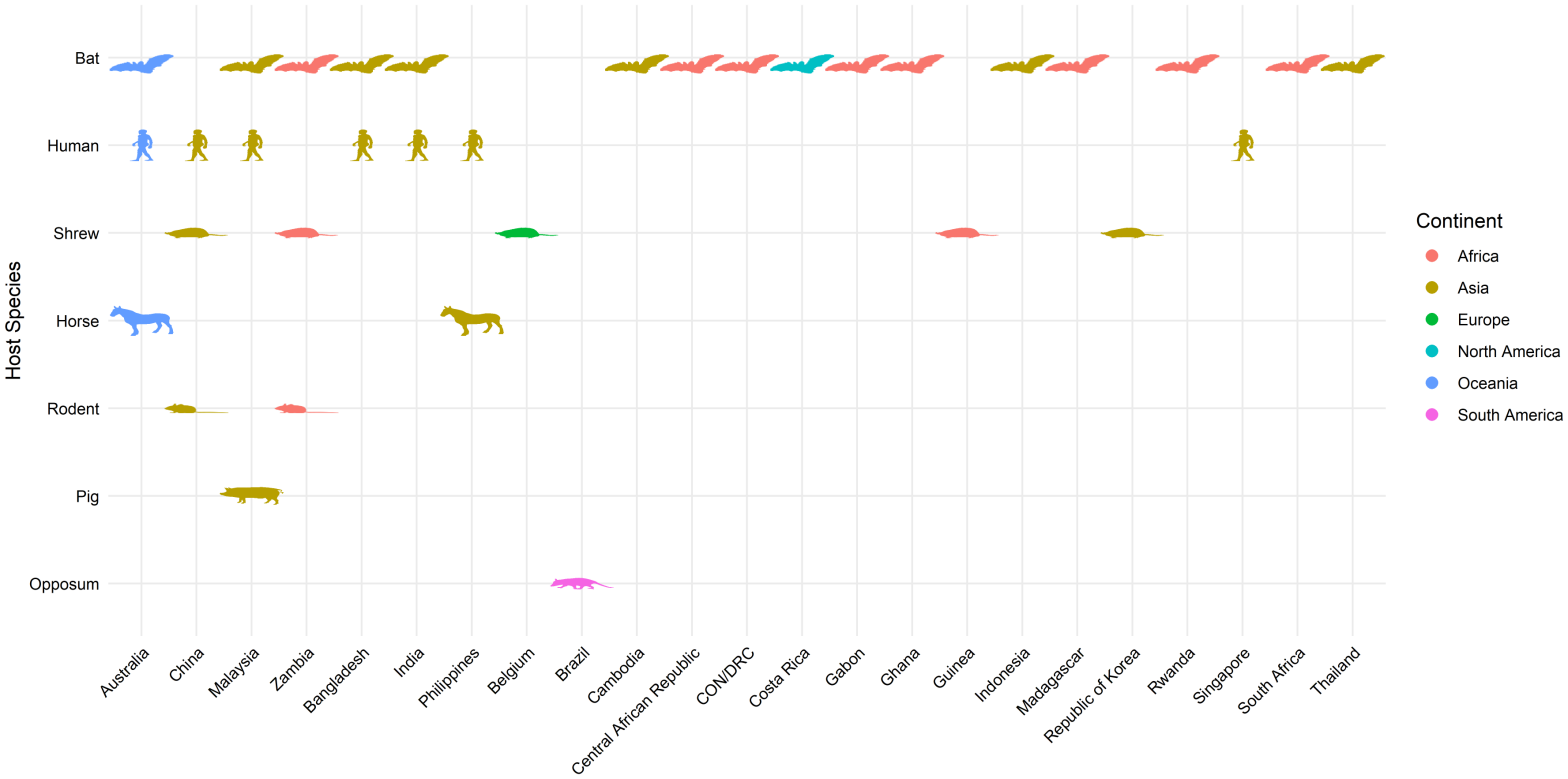
Global locations of henipavirus and henipa-like virus hosts. Map indicates organisms with previously reported infection by henipaviruses and henipa-like viruses.

As a summary, a table is provided showing the geographic locations, case numbers and CFRs of henipavirus and henipa-like virus infections in humans and animals, based on major or representative outbreak events (Supplementary Table S1). Also provided are three figures illustrating the global locations of henipa and henipa-like viruses (Figure 1), outbreaks in humans causing diseases/fatalities (Figure 2) and hosts of these viruses (Figure 3), respectively.

## Discussion

The emergence of henipa-like viruses in new hosts outside the traditionally known endemic regions suggests a tremendous extension of the reservoir diversity and geographic range of the *henipavirus* genus. These new data reinforced the possibility that a much wider range of animal hosts than previously identified may harbor or/and transmit henipa-like viruses. Considering the distinct taxonomic classifications of the currently known hosts, the discovery of additional new hosts would not be surprising, which could further expand the areas under potential threats.

While HeV and NiV, the prototype zoonotic henipaviruses, are among the deadliest pathogens known to humans, the zoonotic and pathogenic potential of the newly detected henipa-like viruses remains largely undetermined. However, their close relation to these inherently deadly zoonotic viruses itself deserves serious attention. Several lines of observations, furthermore, have pointed to the possibility of spillover events of concern. In the Cameroon study mentioned above, antibodies to henipaviruses were detected in 48% and 3–4% of the sampled African Eidolon bats (n = 44) and humans (n = 497), respectively. Human seropositivity was almost exclusively found in those who had butchered bats for meat (Pernet et al., 2014b). This provides evidence suggesting human infections by African henipa-like viruses following spillovers from bats. It is unclear whether these viruses result in disease in infected humans. However, it has been proposed that they might be involved in unrecognized human disease and contribute to the prevalence of unresolved encephalitis cases (Mathers et al., 2007; Hayman et al., 2008; Drexler et al., 2012; Pernet et al., 2014b; Rissanen et al., 2017). Moreover, MojV has a suspected link to fatal pneumonia (Wu et al., 2014). Finally, the correlation between LayV and acute febrile cases has largely been established as causal based on the association of the viral presence, host antibody response, viremia and magnitude of viral load with the disease occurrence, progression and severity (Zhang et al., 2022). An additional note on LayV is that domestic animals such as goats seem to be susceptible to LayV infection, with a conceivable potential to serve as amplification hosts for viral transmission, although it is unknown if they are susceptible to LayV disease (Zhang et al., 2022). These lurking threats deserve urgent further investigation. It should also be stressed that emerging viruses even apparently harmless could rapidly become devastating pathogens, as well exemplified by the Brazilian epidemic of Zika virus, which had been historically thought to be of little or no pathogenicity (Rissanen et al., 2017).

Reservoir hosts of henipaviruses and emerging henipa-like viruses now have an overwhelmingly broad distribution on the planet. Climate change is expected to further increase the emergence of favorable ecological environment leading to the extension of their habitats into new locations (Latinne and Morand, 2022). This will put new areas under the risk of henipavirus disease. Meanwhile, the ever-growing human activities of expansion including unlimited urbanization and excessive farming have been encroaching into wildlife territories. Resulting from these factors, more domestic animals such as the ruminants as well as people live in closer contact with the henipavirus reservoirs, resulting in more zoonotic spillover events (Field, 2009; Kessler et al., 2018; Yuen et al., 2021; Latinne and Morand, 2022; Eby et al., 2023). These are occurring repeatedly almost every year, and right now (bdnews24.com, 2023; outbreaknewstoday.com, 2023; risingbd.com, 2023). With each spillover event, the likelihood of mutation in a henipavirus (RNA virus like SARS-CoV-2) increases (Li et al., 2022), and the virus is in an environment that selects for better adaptation in the new host. As a result, a new mutant strain could arise at any time with more efficient and sustained transmission in livestock and/or humans, which could spark a pandemic with devastating impact on the economy and public health. NiV-B possesses the highest potential to become such a mutant virus among currently known henipaviruses and henipa-like viruses due to a combination of seriously concerning characteristics: highest CFR, human-to-human transmission, respiratory disease and possible respiratory transmission, continuing and frequent outbreaks (in regions with high population densities) and unknown intermediate hosts. Since NiV-B causes a CFR of 70–100% in humans, a mutant strain with a similar CFR and high human-to-human transmissibility would be catastrophic. A highly transmissible henipavirus outbreak will respect no borders in the increasingly globalized and interconnected world and its spread can be facilitated by international travel, transportation and trade. The prevention of such outbreaks at the source undoubtedly requires a thorough understanding of the viral transmission pathways in order to establish targets for minimizing, blocking or eliminating spillover events. Furthermore, during the spread of an epidemic or pandemic following an unfortunate onset, the development of effective, targeted response plans to contain the spread or further to prevent resurgence of infections would be challenging with poor knowledge on the transmission routes involving unidentified animal hosts. Proactive spillover prevention and outbreak preparedness will depend on continued serological and molecular surveillance in wildlife and domestic animals in areas at risk as well as controlled experimental investigation to determine the role of these animals in viral transmission, particularly for example the role of domestic ruminants in NiV-B transmission as proposed above. Experimental inoculation studies in ruminants or other animals should complete a comprehensive and in-depth characterization of the biological and clinical features of henipaviral infection. Concerning the potential role of an animal host in amplifying and transmitting the virus, such studies may reach one of the following findings: Noticeable or severe disease accompanied by high-level viral amplification and shedding, no obvious signs of disease but substantial-level viral amplification and shedding, resistance to infection and disease without viral amplification or shedding, or transitional or mixed phenotypes of these. Notably, a cryptic infection in livestock with high level viral amplification and shedding but no prominent signs of illness, if occurring unrecognized, will pose great risk to farmers, slaughterhouse workers and even household consumers who come into contact with infected animals or contaminated meat products.

The resulting knowledge from these efforts will guide the development of targeted strategies to prevent and contain future henipavirus outbreaks. An extreme idea might be to remove the identified viral reservoirs, which would be practically impossible and disastrous given the large numbers and broad distribution of the reservoir hosts and the importance of these animals to the ecosystem and biodiversity. It should be noted that loss of biodiversity often leads to increased disease transmission (Keesing et al., 2010; Drexler et al., 2012). Under the One Health approach, ecological communities harboring the natural reservoir hosts should be treated with conservation and respect rather than interference or destruction. Alternatively, human activities such as urban constructions and agricultural developments should follow optimized design strategies to avoid or minimize human and livestock contact with reservoir hosts. Furthermore, vaccination of livestock populations at risk is a conceivably feasible and effective solution, expected to prevent both disease in livestock and further transmission from intermediate hosts to humans. Vaccination of humans is also anticipated to provide protection against transmission from infected reservoir hosts, intermediate hosts, or humans.

On a final, encouraging note, although the emergence of new henipa-like viruses signifies new challenges, it also provides new opportunities. Different characteristics among closely related viruses can be utilized in comparative functional investigations to understand aspects of henipavirus biology such as evolution, host tropism, cross-species transmission and pathogenicity. So far CedV (Marsh et al., 2012; Lieu et al., 2015; Laing et al., 2018, 2019; Schountz et al., 2019; Yeo et al., 2021), KV (Pernet et al., 2014a; Lee et al., 2015) and MojV (Rissanen et al., 2017; Cheliout Da Silva et al., 2021) have been involved in a number of such studies. Non-pathogenic henipa-like viruses such as CedV could also be used as immunogen vectors in vaccines against pathogenic henipavirus disease, since antigenic overlap could induce cross-reactivity between closely related viruses. Following this idea, we found reports of vaccine development based on recombinant measles virus and recombinant Newcastle disease virus expressing NiV G protein (Kong et al., 2012; Yoneda et al., 2013). Note that these two viruses belong to the same family (*Paramyxoviridae*) as but different genera (*Morbillivirus* and *Avulavirus*, respectively) from henipaviruses. In comparison, a vaccine vector derived from CedV is expected to elicit better cross-protection due to higher extent of antigenic overlap within the *henipavirus* genus. Our previous study suggested that the soluble G protein vaccine was not effective in inducing cellular immunity (Pickering et al., 2016). We propose that CedV as a viral vector could possibly enhance the vaccine efficacy by strengthening the cellular arm of immune protection. Recombinant CedVs displaying G and F proteins of HeV or NiV (Doyle et al., 2021) are anticipated to be exceptional candidate vaccines for testing in livestock and humans.

## Author contributions

HL and BP contributed to the conception and design of the review. HL wrote the first draft of the manuscript. J-YK generated Figures 1–3 and Supplementary Table S1. BP reviewed and edited the manuscript, acquired funding, and supervised the work. All authors contributed to the manuscript revision, read, and approved the submitted version.

## Funding

This work was supported by funding from Canadian Food Inspection Agency.

## Conflict of interest

The authors declare that the research was conducted in the absence of any commercial or financial relationships that could be construed as a potential conflict of interest.

## Publisher’s note

All claims expressed in this article are solely those of the authors and do not necessarily represent those of their affiliated organizations, or those of the publisher, the editors and the reviewers. Any product that may be evaluated in this article, or claim that may be made by its manufacturer, is not guaranteed or endorsed by the publisher.

## Supplementary material

The Supplementary material for this article can be found online at: https://www.frontiersin.org/articles/10.3389/fmicb.2023.1167085/full#supplementary-material

## References

[ref1] Abdullah S. Tan C. T. (2014). Henipavirus encephalitis. Handb. Clin. Neurol. 123, 663–670. doi: 10.1016/B978-0-444-53488-0.00032-8 25015510

[ref2] Amarasinghe G. K. Bào Y. Basler C. F. Bavari S. Beer M. Bejerman N.. (2017). Taxonomy of the order Mononegavirales: update 2017. Arch. Virol. 162, 2493–2504. doi: 10.1007/s00705-017-3311-7, PMID: 28389807 PMC5831667

[ref3] Amaya M. Cheng H. Borisevich V. Navaratnarajah C. K. Cattaneo R. Cooper L.. (2021). A recombinant cedar virus based high-throughput screening assay for henipavirus antiviral discovery. Antivir. Res. 193:105084. doi: 10.1016/j.antiviral.2021.105084, PMID: 34077807 PMC8631057

[ref4] Annand E. J. Horsburgh B. A. Xu K. Reid P. A. Poole B. de Kantzow M. C.. (2022). Novel Hendra virus variant detected by sentinel surveillance of horses in Australia. Emerg. Infect. Dis. 28, 693–704. doi: 10.3201/eid2803.211245, PMID: 35202527 PMC8888208

[ref5] Arunkumar G. Abdulmajeed J. Santhosha D. Aswathyraj S. Robin S. Jayaram A.. (2019). Persistence of Nipah virus RNA in semen of survivor. Clin. Infect. Dis. 69, 377–378. doi: 10.1093/cid/ciy1092, PMID: 30590474

[ref6] Banglapedia (2023). Livestock. Available at: https://en.banglapedia.org/index.php/Livestock (Accessed February 10, 2023).

[ref7] bdnews24.com (2023). Bangladesh reports two new Nipah virus deaths. Available at: https://bdnews24.com/health/03lrm6ebbo (Accessed February 10, 2023).

[ref8] Broder C. C. Xu K. Nikolov D. B. Zhu Z. Dimitrov D. S. Middleton D.. (2013). A treatment for and vaccine against the deadly Hendra and Nipah viruses. Antivir. Res. 100, 8–13. doi: 10.1016/j.antiviral.2013.06.012, PMID: 23838047 PMC4418552

[ref9] Bruno L. Nappo M. A. Ferrari L. di Lecce R. Guarnieri C. Cantoni A. M.. (2023). Nipah virus disease: epidemiological, clinical, diagnostic and legislative aspects of this unpredictable emerging zoonosis. Animals (Basel) 13:159. doi: 10.3390/ani13010159, PMID: 36611767 PMC9817766

[ref10] CDC (1999a). Outbreak of Hendra-like virus--Malaysia and Singapore, 1998-1999. MMWR Morb. Mortal. Wkly Rep. 48, 265–269. 10227800

[ref11] CDC (1999b). Update: outbreak of Nipah virus--Malaysia and Singapore, 1999. MMWR Morb. Mortal. Wkly Rep. 48, 335–337. 10366143

[ref12] Chadha M. S. Comer J. A. Lowe L. Rota P. A. Rollin P. E. Bellini W. J.. (2006). Nipah virus-associated encephalitis outbreak, Siliguri, India. Int. Conf. Emerg. Infect. Dis. 12, 235–240. doi: 10.3201/eid1202.051247, PMID: 16494748 PMC3373078

[ref13] Cheliout Da Silva S. Yan L. Dang H. V. Xu K. Epstein J. H. Veesler D.. (2021). Functional analysis of the fusion and attachment glycoproteins of Mojiang Henipavirus. Viruses 13:517. doi: 10.3390/v13030517, PMID: 33809833 PMC8004131

[ref14] Ching P. K. de los Reyes V. C. Sucaldito M. N. Tayag E. Columna-Vingno A. B. Malbas F. F. Jr.. (2015). Outbreak of henipavirus infection, Philippines, 2014. Emerg. Infect. Dis. 21, 328–331. doi: 10.3201/eid2102.141433, PMID: 25626011 PMC4313660

[ref15] Chong H. T. Kunjapan S. R. Thayaparan T. Geok Tong J. M. Petharunam V. Jusoh M. R.. (2002). Nipah encephalitis outbreak in Malaysia, clinical features in patients from Seremban. Can. J. Neurol. Sci. 29, 83–87. doi: 10.1017/s0317167100001785, PMID: 11858542

[ref16] Chowdhury S. Khan S. U. Crameri G. Epstein J. H. Broder C. C. Islam A.. (2014). Serological evidence of henipavirus exposure in cattle, goats and pigs in Bangladesh. PLoS Negl. Trop. Dis. 8:e3302. doi: 10.1371/journal.pntd.0003302, PMID: 25412358 PMC4238985

[ref17] Chua K. B. (2003). Nipah virus outbreak in Malaysia. J. Clin. Virol. 26, 265–275. doi: 10.1016/s1386-6532(02)00268-8 12637075

[ref18] Chua K. B. Bellini W. J. Rota P. A. Harcourt B. H. Tamin A. Lam S. K.. (2000). Nipah virus: a recently emergent deadly paramyxovirus. Science 288, 1432–1435. doi: 10.1126/science.288.5470.1432, PMID: 10827955

[ref19] Chua K. B. Goh K. J. Wong K. T. Kamarulzaman A. Tan P. S. Ksiazek T. G.. (1999). Fatal encephalitis due to Nipah virus among pig-farmers in Malaysia. Lancet 354, 1257–1259. doi: 10.1016/S0140-6736(99)04299-3 10520635

[ref20] Cortes M. C. Cauchemez S. Lefrancq N. Luby S. P. Jahangir Hossain M. Sazzad H. M. S.. (2018). Characterization of the spatial and temporal distribution of Nipah virus spillover events in Bangladesh, 2007–2013. J. Infect. Dis. 217, 1390–1394. doi: 10.1093/infdis/jiy015, PMID: 29351657 PMC5894074

[ref21] de Araujo J. Lo M. K. Tamin A. Ometto T. L. Thomazelli L. M. Nardi M. S.. (2017). Antibodies Against Henipa-Like Viruses in Brazilian Bats. Vector Borne Zoonotic Dis. 17, 271–274. doi: 10.1089/vbz.2016.2051, PMID: 28103156

[ref22] de Wit E. Prescott J. Falzarano D. Bushmaker T. Scott D. Feldmann H.. (2014). Foodborne transmission of nipah virus in Syrian hamsters. PLoS Pathog. 10:e1004001. doi: 10.1371/journal.ppat.1004001, PMID: 24626480 PMC3953481

[ref23] Doyle M. P. Kose N. Borisevich V. Binshtein E. Amaya M. Nagel M.. (2021). Cooperativity mediated by rationally selected combinations of human monoclonal antibodies targeting the henipavirus receptor binding protein. Cell Rep. 36:109628. doi: 10.1016/j.celrep.2021.109628, PMID: 34469726 PMC8527959

[ref24] Drexler J. F. Corman V. M. Gloza-Rausch F. Seebens A. Annan A. Ipsen A.. (2009). Henipavirus RNA in African bats. PLoS One 4:e6367. doi: 10.1371/journal.pone.0006367 19636378 PMC2712088

[ref25] Drexler J. F. Corman V. M. Müller M. A. Maganga G. D. Vallo P. Binger T.. (2012). Bats host major mammalian paramyxoviruses. Nat. Commun. 3:796. doi: 10.1038/ncomms1796, PMID: 22531181 PMC3343228

[ref26] Eaton B. T. Broder C. C. Middleton D. Wang L. F. (2006). Hendra and Nipah viruses: different and dangerous. Nat. Rev. Microbiol. 4, 23–35. doi: 10.1038/nrmicro1323 16357858 PMC7097447

[ref27] Eby P. Peel A. J. Hoegh A. Madden W. Giles J. R. Hudson P. J.. (2023). Pathogen spillover driven by rapid changes in bat ecology. Nature 613, 340–344. doi: 10.1038/s41586-022-05506-2, PMID: 36384167 PMC9768785

[ref28] Field H. E. (2009). Bats and emerging zoonoses: henipaviruses and SARS. Zoonoses Public Health 56, 278–284. doi: 10.1111/j.1863-2378.2008.01218.xJVB1218 [pii] 19497090

[ref29] Field H. Jordan D. Edson D. Morris S. Melville D. Parry-Jones K.. (2015). Spatiotemporal aspects of Hendra virus infection in pteropid bats (flying-foxes) in eastern Australia. PLoS One 10:e0144055. doi: 10.1371/journal.pone.0144055, PMID: 26625128 PMC4666458

[ref30] Gazal S. Sharma N. Gazal S. Tikoo M. Shikha D. Badroo G. A.. (2022). Nipah and Hendra viruses: deadly zoonotic paramyxoviruses with the potential to cause the next pandemic. Pathogens 11:1419. doi: 10.3390/pathogens11121419, PMID: 36558753 PMC9784551

[ref31] Geisbert T. W. Feldmann H. Broder C. C. (2012). Animal challenge models of henipavirus infection and pathogenesis. Curr. Top. Microbiol. Immunol. 359, 153–177. doi: 10.1007/82_2012_208 22476556 PMC7120132

[ref32] Goh K. J. Tan C. T. Chew N. K. Tan P. S. Kamarulzaman A. Sarji S. A.. (2000). Clinical features of Nipah virus encephalitis among pig farmers in Malaysia. N. Engl. J. Med. 342, 1229–1235. doi: 10.1056/NEJM200004273421701, PMID: 10781618

[ref33] Gurley E. S. Hegde S. T. Hossain K. Sazzad H. M. S. Hossain M. J. Rahman M.. (2017). Convergence of humans, bats, trees, and culture in Nipah virus transmission, Bangladesh. Int. Conf. Emerg. Infect. Dis. 23, 1446–1453. doi: 10.3201/eid2309.161922, PMID: 28820130 PMC5572889

[ref34] Gurley E. S. Montgomery J. M. Hossain M. J. Bell M. Azad A. K. Islam M. R.. (2007). Person-to-person transmission of Nipah virus in a Bangladeshi community. Emerg. Infect. Dis. 13, 1031–1037. doi: 10.3201/eid1307.061128, PMID: 18214175 PMC2878219

[ref35] Halpin K. Hyatt A. D. Fogarty R. Middleton D. Bingham J. Epstein J. H.. (2011). Pteropid bats are confirmed as the reservoir hosts of henipaviruses: a comprehensive experimental study of virus transmission. Am. J. Trop. Med. Hyg. 85, 946–951. doi: 10.4269/ajtmh.2011.10-0567, PMID: 22049055 PMC3205647

[ref36] Halpin K. Rota P. (2014). A review of Hendra virus and Nipah virus infections in man and other animals. Zoonoses 997–1012. doi: 10.1007/978-94-017-9457-2_40

[ref37] Hayman D. T. Suu-Ire R. Breed A. C. McEachern J. A. Wang L. Wood J. L.. (2008). Evidence of henipavirus infection in West African fruit bats. PLoS One 3:e2739. doi: 10.1371/journal.pone.0002739, PMID: 18648649 PMC2453319

[ref38] Hernández L. H. A. da Paz T. Y. B. Silva S. P. D. Silva F. S. D. Barros B. C. V. Nunes B. T. D.. (2022). First genomic evidence of a Henipa-like virus in Brazil. Viruses 14:2167. doi: 10.3390/v14102167, PMID: 36298723 PMC9608811

[ref39] Hsu V. P. Hossain M. J. Parashar U. D. Ali M. M. Ksiazek T. G. Kuzmin I.. (2004). Nipah virus encephalitis reemergence, Bangladesh. Int. Conf. Emerg. Infect. Dis. 10, 2082–2087. doi: 10.3201/eid1012.040701, PMID: 15663842 PMC3323384

[ref40] ICTV (2023). Genus: Henipavirus. Available at: https://ictv.global/report/chapter/paramyxoviridae/paramyxoviridae/henipavirus (Accessed February 11, 2023).

[ref41] Iehlé C. Razafitrimo G. Razainirina J. Andriaholinirina N. Goodman S. M. Faure C.. (2007). Henipavirus and Tioman virus antibodies in Pteropodid bats, Madagascar. Int. Conf. Emerg. Infect. Dis. 13, 159–161. doi: 10.3201/eid1301.060791, PMID: 17370536 PMC2725826

[ref42] Kasloff S. B. Leung A. Pickering B. S. Smith G. Moffat E. Collignon B.. (2019). Pathogenicity of Nipah henipavirus Bangladesh in a swine host. Sci. Rep. 9:5230. doi: 10.1038/s41598-019-40476-y, PMID: 30914663 PMC6435791

[ref43] Keesing F. Belden L. K. Daszak P. Dobson A. Harvell C. D. Holt R. D.. (2010). Impacts of biodiversity on the emergence and transmission of infectious diseases. Nature 468, 647–652. doi: 10.1038/nature09575 21124449 PMC7094913

[ref44] Kessler M. K. Becker D. J. Peel A. J. Justice N. V. Lunn T. Crowley D. E.. (2018). Changing resource landscapes and spillover of henipaviruses. Ann. N. Y. Acad. Sci. 1429, 78–99. doi: 10.1111/nyas.13910, PMID: 30138535 PMC6778453

[ref45] Khan S. U. Gurley E. S. Hossain M. J. Nahar N. Sharker M. A. Luby S. P. (2012). A randomized controlled trial of interventions to impede date palm sap contamination by bats to prevent nipah virus transmission in Bangladesh. PLoS One 7:e42689. doi: 10.1371/journal.pone.0042689 22905160 PMC3414453

[ref46] Kong D. Wen Z. Su H. Ge J. Chen W. Wang X.. (2012). Newcastle disease virus-vectored Nipah encephalitis vaccines induce B and T cell responses in mice and long-lasting neutralizing antibodies in pigs. Virology 432, 327–335. doi: 10.1016/j.virol.2012.06.001, PMID: 22726244

[ref47] Kummer S. Kranz D. C. (2022). Henipaviruses-a constant threat to livestock and humans. PLoS Negl. Trop. Dis. 16:e0010157. doi: 10.1371/journal.pntd.0010157PNTD-D-21-00815 [pii] 35180217 PMC8856525

[ref48] Laing E. D. Amaya M. Navaratnarajah C. K. Feng Y. R. Cattaneo R. Wang L. F.. (2018). Rescue and characterization of recombinant cedar virus, a non-pathogenic Henipavirus species. Virol. J. 15:56. doi: 10.1186/s12985-018-0964-0, PMID: 29587789 PMC5869790

[ref49] Laing E. D. Navaratnarajah C. K. Cheliout da Silva S. Petzing S. R. Xu Y. Sterling S. L.. (2019). Structural and functional analyses reveal promiscuous and species specific use of ephrin receptors by cedar virus. Proc. Natl. Acad. Sci. U. S. A. 116, 20707–20715. doi: 10.1073/pnas.1911773116, PMID: 31548390 PMC6789926

[ref50] Lam S. K. Chua K. B. (2002). Nipah virus encephalitis outbreak in Malaysia. Clin. Infect. Dis. 34, S48–S51. doi: 10.1086/338818 11938496

[ref51] Latinne A. Morand S. (2022). Climate anomalies and spillover of bat-borne viral diseases in the Asia-Pacific region and the Arabian peninsula. Viruses 14:1100. doi: 10.3390/v14051100 35632842 PMC9145311

[ref52] Lee S. H. Kim K. Kim J. No J. S. Park K. Budhathoki S.. (2021). Discovery and genetic characterization of novel paramyxoviruses related to the genus Henipavirus in Crocidura species in the Republic of Korea. Viruses 13:2020. doi: 10.3390/v13102020, PMID: 34696450 PMC8537881

[ref53] Lee B. Pernet O. Ahmed A. A. Zeltina A. Beaty S. M. Bowden T. A. (2015). Molecular recognition of human ephrinB2 cell surface receptor by an emergent African henipavirus. Proc. Natl. Acad. Sci. U. S. A. 112, E2156–E2165. doi: 10.1073/pnas.1501690112 25825759 PMC4418902

[ref54] Lewis C. E. Pickering B. (2022). Livestock and risk group 4 pathogens: researching zoonotic threats to public health and agriculture in maximum containment. ILAR J. 61, 86–102. doi: 10.1093/ilar/ilab029 34864994 PMC8759435

[ref55] Li H. Bello A. Smith G. Kielich D. M. S. Strong J. E. Pickering B. S. (2022). Degenerate sequence-based CRISPR diagnostic for Crimean-Congo hemorrhagic fever virus. PLoS Negl. Trop. Dis. 16:e0010285. doi: 10.1371/journal.pntd.0010285 PNTD-D-21-00739 [pii] 35271569 PMC8939784

[ref56] Lieu K. G. Marsh G. A. Wang L. F. Netter H. J. (2015). The non-pathogenic Henipavirus cedar paramyxovirus phosphoprotein has a compromised ability to target STAT1 and STAT2. Antivir. Res. 124, 69–76. doi: 10.1016/j.antiviral.2015.09.017 26526590

[ref57] Lo M. K. Lowe L. Hummel K. B. Sazzad H. M. Gurley E. S. Hossain M. J.. (2012). Characterization of Nipah virus from outbreaks in Bangladesh, 2008–2010. Emerg. Infect. Dis. 18, 248–255. doi: 10.3201/eid1802.111492, PMID: 22304936 PMC3310473

[ref58] Luby S. P. Gurley E. S. (2012). Epidemiology of henipavirus disease in humans. Curr. Top. Microbiol. Immunol. 359, 25–40. doi: 10.1007/82_2012_207 22752412

[ref59] Luby S. P. Gurley E. S. Hossain M. J. (2009a). Transmission of human infection with Nipah virus. Clin. Infect. Dis. 49, 1743–1748. doi: 10.1086/647951 19886791 PMC2784122

[ref60] Luby S. P. Hossain M. J. Gurley E. S. Ahmed B. N. Banu S. Khan S. U.. (2009b). Recurrent zoonotic transmission of Nipah virus into humans, Bangladesh, 2001-2007. Emerg. Infect. Dis. 15, 1229–1235. doi: 10.3201/eid1508.081237 19751584 PMC2815955

[ref61] Madera S. Kistler A. Ranaivoson H. C. Ahyong V. Andrianiaina A. Andry S.. (2022). Discovery and genomic characterization of a novel Henipavirus, Angavokely virus, from fruit bats in Madagascar. J. Virol. 96:e0092122. doi: 10.1128/jvi.00921-22, PMID: 36040175 PMC9517717

[ref62] Mallapaty S. (2022). New 'Langya' virus identified in China: what scientists know so far. Nature 608, 656–657. doi: 10.1038/d41586-022-02175-z 35953571

[ref63] Marsh G. A. de Jong C. Barr J. A. Tachedjian M. Smith C. Middleton D.. (2012). Cedar virus: a novel Henipavirus isolated from Australian bats. PLoS Pathog. 8:e1002836. doi: 10.1371/journal.ppat.1002836, PMID: 22879820 PMC3410871

[ref64] Martin G. Yanez-Arenas C. Plowright R. K. Chen C. Roberts B. Skerratt L. F. (2018). Hendra virus spillover is a bimodal system driven by climatic factors. EcoHealth 15, 526–542. doi: 10.1007/s10393-017-1309-y 29349533

[ref65] Mathers C. D. Ezzati M. Lopez A. D. (2007). Measuring the burden of neglected tropical diseases: the global burden of disease framework. PLoS Negl. Trop. Dis. 1:e114. doi: 10.1371/journal.pntd.0000114 18060077 PMC2100367

[ref66] McKee C. D. Islam A. Luby S. P. Salje H. Hudson P. J. Plowright R. K.. (2021). The ecology of Nipah virus in Bangladesh: a Nexus of land-use change and opportunistic feeding behavior in bats. Viruses 13:169. doi: 10.3390/v13020169, PMID: 33498685 PMC7910977

[ref67] Mehand M. S. Millett P. Al-Shorbaji F. Roth C. Kieny M. P. Murgue B. (2018). World health organization methodology to prioritize emerging infectious diseases in need of research and development. Emerg. Infect. Dis. 24:e171427. doi: 10.3201/eid2409.171427 30124424 PMC6106429

[ref68] Middleton D. J. Weingartl H. M. (2012). Henipaviruses in their natural animal hosts. Curr. Top. Microbiol. Immunol. 359, 105–121. doi: 10.1007/82_2012_210 22476529

[ref69] Mohd Nor M. N. Gan C. H. Ong B. L. (2000). Nipah virus infection of pigs in peninsular Malaysia. Rev. Sci. Tech. 19, 160–165. doi: 10.20506/rst.19.1.1202 11189713

[ref70] Murray K. Selleck P. Hooper P. Hyatt A. Gould A. Gleeson L.. (1995). A morbillivirus that caused fatal fisease in horses and humans. Science 268, 94–97. doi: 10.1126/science.7701348 7701348

[ref71] Nahar N. Mondal U. K. Hossain M. J. Khan M. S. Sultana R. Gurley E. S.. (2014). Piloting the promotion of bamboo skirt barriers to prevent Nipah virus transmission through date palm sap in Bangladesh. Glob. Health Promot. 21, 7–15. doi: 10.1177/1757975914528249, PMID: 24755262 PMC4666517

[ref72] Nikolay B. Salje H. Hossain M. J. Khan A. Sazzad H. M. S. Rahman M.. (2019). Transmission of Nipah virus - 14 years of investigations in Bangladesh. N. Engl. J. Med. 380, 1804–1814. doi: 10.1056/NEJMoa1805376, PMID: 31067370 PMC6547369

[ref73] Nordin M. N. (1999). Nipah DIsease in Maysia. OIE Dis. Inf. 12:20.

[ref74] nsw.gov.au (2023). Summary of human cases of Hendra virus infection. Available at: https://www.health.nsw.gov.au/Infectious/controlguideline/Pages/hendra-case-summary.aspx (Accessed February 10, 2023).

[ref75] outbreaknewstoday.com (2023). Bangladesh Nipah virus update: 10 cases and 7 deaths in 2023. Available at: http://outbreaknewstoday.com/bangladesh-nipah-virus-update-10-cases-and-7-deaths-in-2023/ (Accessed February 11, 2023).

[ref76] Paton N. I. Leo Y. S. Zaki S. R. Auchus A. P. Lee K. E. Ling A. E.. (1999). Outbreak of Nipah-virus infection among abattoir workers in Singapore. Lancet 354, 1253–1256. doi: 10.1016/S0140-6736(99)04379-2, PMID: 10520634

[ref77] Peel A. J. Yinda C. K. Annand E. J. Dale A. S. Eby P. Eden J. S.. (2022). Novel Hendra virus variant circulating in black flying foxes and grey-headed flying foxes, Australia. Int. Conf. Emerg. Infect. Dis. 28, 1043–1047. doi: 10.3201/eid2805.212338, PMID: 35447052 PMC9045453

[ref78] Pernet O. Beaty S. Lee B. (2014a). Functional rectification of the newly described African henipavirus fusion glycoprotein (Gh-M74a). J. Virol. 88, 5171–5176. doi: 10.1128/JVI.03655-13 24522929 PMC3993810

[ref79] Pernet O. Schneider B. S. Beaty S. M. LeBreton M. Yun T. E. Park A.. (2014b). Evidence for henipavirus spillover into human populations in Africa. Nat. Commun. 5:5342. doi: 10.1038/ncomms6342, PMID: 25405640 PMC4237230

[ref80] pib.gov.in (2023). Livestock census. Available at: https://pib.gov.in/PressReleaseIframePage.aspx?PRID=1813802 (Accessed February 10, 2023).

[ref81] Pickering B. S. Hardham J. M. Smith G. Weingartl E. T. Dominowski P. J. Foss D. L.. (2016). Protection against henipaviruses in swine requires both, cell-mediated and humoral immune response. Vaccine 34, 4777–4786. doi: 10.1016/j.vaccine.2016.08.028, PMID: 27544586 PMC6161494

[ref82] Playford E. G. McCall B. Smith G. Slinko V. Allen G. Smith I.. (2010). Human Hendra virus encephalitis associated with equine outbreak, Australia, 2008. Emerg. Infect. Dis. 16, 219–223. doi: 10.3201/eid1602.090552, PMID: 20113550 PMC2957996

[ref83] qld.gov.au (2023). Summary of Hendra virus incidents in horses. Available from: https://www.business.qld.gov.au/industries/service-industries-professionals/service-industries/veterinary-surgeons/guidelines-hendra/incident-summary (Accessed February 10, 2023).

[ref84] Quarleri J. Galvan V. Delpino M. V. (2022). Henipaviruses: an expanding global public health concern? Geroscience. 44, 2447–2459. doi: 10.1007/s11357-022-00670-9 36219280 PMC9550596

[ref85] Rahman M. A. Hossain M. J. Sultana S. Homaira N. Khan S. U. Rahman M.. (2012). Date palm sap linked to Nipah virus outbreak in Bangladesh, 2008. Vector Borne Zoonotic Dis. 12, 65–72. doi: 10.1089/vbz.2011.0656, PMID: 21923274

[ref86] Reynes J. M. Counor D. Ong S. Faure C. Seng V. Molia S.. (2005). Nipah virus in Lyle's flying foxes, Cambodia. Int. Conf. Emerg. Infect. Dis. 11, 1042–1047. doi: 10.3201/eid1107.041350, PMID: 16022778 PMC3371782

[ref87] Rima B. Balkema-Buschmann A. Dundon W. G. Duprex P. Easton A. Fouchier R.. (2019). ICTV virus taxonomy profile: Paramyxoviridae. J. Gen. Virol. 100, 1593–1594. doi: 10.1099/jgv.0.001328 31609197 PMC7273325

[ref88] risingbd.com (2023). Nipah virus claims 5 lives this year: home minister. Available at: https://www.risingbd.com/english/national/news/93117 (Accessed February 11, 2023).

[ref89] Rissanen I. Ahmed A. A. Azarm K. Beaty S. Hong P. Nambulli S.. (2017). Idiosyncratic Mòjiāng virus attachment glycoprotein directs a host-cell entry pathway distinct from genetically related henipaviruses. Nat. Commun. 8:16060. doi: 10.1038/ncomms16060, PMID: 28699636 PMC5510225

[ref90] Sasaki M. Muleya W. Ishii A. Orba Y. Hang’ombe B. M. Mweene A. S.. (2014). Molecular epidemiology of paramyxoviruses in Zambian wild rodents and shrews. J. Gen. Virol. 95, 325–330. doi: 10.1099/vir.0.058404-0, PMID: 24189618

[ref91] Schountz T. Campbell C. Wagner K. Rovnak J. Martellaro C. DeBuysscher B. L.. (2019). Differential innate immune responses elicited by Nipah virus and cedar virus correlate with disparate in vivo pathogenesis in hamsters. Viruses 11:219. doi: 10.3390/v11030291 30909389 PMC6466075

[ref92] Skowron K. Bauza-Kaszewska J. Grudlewska-Buda K. Wiktorczyk-Kapischke N. Zacharski M. Bernaciak Z.. (2021). Nipah virus-another threat from the world of zoonotic viruses. Front. Microbiol. 12:811157. doi: 10.3389/fmicb.2021.811157, PMID: 35145498 PMC8821941

[ref93] Sweileh W. M. (2017). Global research trends of World Health Organization's top eight emerging pathogens. Glob. Health 13:9. doi: 10.1186/s12992-017-0233-9 [pii] PMC5299748 28179007

[ref94] Taylor J. Thompson K. Annand E. J. Massey P. D. Bennett J. Eden J. S.. (2022). Novel variant Hendra virus genotype 2 infection in a horse in the greater Newcastle region, New South Wales, Australia. One Health 15:100423. doi: 10.1016/j.onehlt.2022.100423, PMID: 36277112 PMC9582560

[ref95] Temmam S. Vongphayloth K. Baquero E. Munier S. Bonomi M. Regnault B.. (2022). Bat coronaviruses related to SARS-CoV-2 and infectious for human cells. Nature 604, 330–336. doi: 10.1038/s41586-022-04532-4, PMID: 35172323

[ref96] thedailystar.net (2023). One dies of Nipah virus at DMCH. Available at: https://www.thedailystar.net/health/disease/coronavirus/events-who/deaths-infections/news/1-covid-19-death-24-hours-positivity-rate-045-3246631 (Accessed February 15, 2023)

[ref97] Tian J. Sun J. Li D. Wang N. Wang L. Zhang C.. (2022). Emerging viruses: Cross-species transmission of coronaviruses, filoviruses, henipaviruses, and rotaviruses from bats. Cell Rep. 39:110969. doi: 10.1016/j.celrep.2022.110969, PMID: 35679864 PMC9148931

[ref98] Vanmechelen B. Meurs S. Horemans M. Loosen A. Joly Maes T. Laenen L.. (2022). The characterization of multiple novel paramyxoviruses highlights the diverse nature of the subfamily *Orthoparamyxovirinae*. Virus Evol. 8:veac061. doi: 10.1093/ve/veac061, PMID: 35854826 PMC9290864

[ref99] Wang J. Anderson D. E. Halpin K. Hong X. Chen H. Walker S.. (2021). A new Hendra virus genotype found in Australian flying foxes. Virol. J. 18:197. doi: 10.1186/s12985-021-01652-7, PMID: 34641882 PMC8510678

[ref100] Wang Z. Dang H. V. Amaya M. Xu Y. Yin R. Yan L.. (2022). Potent monoclonal antibody-mediated neutralization of a divergent Hendra virus variant. Proc. Natl. Acad. Sci. U. S. A. 119:e2122769119. doi: 10.1073/pnas.2122769119, PMID: 35617431 PMC9295758

[ref101] Weingartl H. M. Berhane Y. Czub M. (2009). Animal models of henipavirus infection: a review. Vet. J. 181, 211–220. doi: 10.1016/j.tvjl.2008.10.016 S1090-0233(08)00376-6 [pii] 19084436

[ref102] WHO (2021). Nipah virus disease - India. Available at: https://www.who.int/emergencies/disease-outbreak-news/item/nipah-virus-disease---india (Accessed February 10, 2023).

[ref103] WHO (2023a). Prioritizing diseases for research and development in emergency contexts. Available at: https://www.who.int/activities/prioritizing-diseases-for-research-and-development-in-emergency-contexts (Accessed February 11, 2023)

[ref104] WHO (2023b). R&D Blueprint. Available at: https://www.who.int/teams/blueprint (Accessed February 11, 2023).

[ref105] WHO (2023c). WHO to identify pathogens that could cause future outbreaks and pandemics. Geneva World Health Organization

[ref106] Wong K. T. Ong K. C. (2011). Pathology of acute henipavirus infection in humans and animals. Pathol. Res. Int. 2011:567248. doi: 10.4061/2011/567248 PMC3180787 21961078

[ref107] Wong K. T. Tan C. T. (2012). Clinical and pathological manifestations of human henipavirus infection. Curr. Top. Microbiol. Immunol. 359, 95–104. doi: 10.1007/82_2012_205 22427144

[ref108] Wu Z. Yang L. Yang F. Ren X. Jiang J. Dong J.. (2014). Novel Henipa-like virus, Mojiang Paramyxovirus, in rats, China, 2012. Emerg. Infect. Dis. 20, 1064–1066. doi: 10.3201/eid2006.131022, PMID: 24865545 PMC4036791

[ref109] Yadav P. D. Sahay R. R. Balakrishnan A. Mohandas S. Radhakrishnan C. Gokhale M. D.. (2022). Nipah virus outbreak in Kerala state, India amidst of COVID-19 pandemic. Front. Public Health 10:818545. doi: 10.3389/fpubh.2022.818545, PMID: 35252095 PMC8891450

[ref110] Yeo Y. Y. Buchholz D. W. Gamble A. Jager M. Aguilar H. C. (2021). Headless Henipaviral receptor binding glycoproteins reveal fusion modulation by the head/stalk Interface and post-receptor binding contributions of the head domain. J. Virol. 95:e0066621. doi: 10.1128/JVI.00666-21 34288734 PMC8475510

[ref111] Yoneda M. Georges-Courbot M. C. Ikeda F. Ishii M. Nagata N. Jacquot F.. (2013). Recombinant measles virus vaccine expressing the Nipah virus glycoprotein protects against lethal Nipah virus challenge. PLoS One 8:e58414. doi: 10.1371/journal.pone.0058414, PMID: 23516477 PMC3597623

[ref112] Yuen K. Y. Fraser N. S. Henning J. Halpin K. Gibson J. S. Betzien L.. (2021). Hendra virus: epidemiology dynamics in relation to climate change, diagnostic tests and control measures. One Health 12:100207. doi: 10.1016/j.onehlt.2020.100207, PMID: 33363250 PMC7750128

[ref113] Zhang X. A. Li H. Jiang F. C. Zhu F. Zhang Y. F. Chen J. J.. (2022). A zoonotic Henipavirus in febrile patients in China. N. Engl. J. Med. 387, 470–472. doi: 10.1056/NEJMc2202705, PMID: 35921459

